# Associations between the structural and functional aspects of social relations and poor mental health: a cross-sectional register study

**DOI:** 10.1186/s12889-017-4871-x

**Published:** 2017-11-03

**Authors:** Lise Røntved Hansen, Stinna Bibi Pedersen, Charlotte Overgaard, Christian Torp-Pedersen, Line Rosenkilde Ullits

**Affiliations:** 1 Public Health and Epidemiology Group, Department of Health and Science and Technology, Aalborg University, Niels Jernes vej 12, -9220 Aalborg, DK Denmark; 2 Department of Clinical Epidemiology, Aalborg University Hospital, Sdr. Skovvej 15, 9000 Aalborg, Denmark

**Keywords:** Mental health, Social relations, Structural, Functional, Social network

## Abstract

**Background:**

Social relations influence mental health through different pathways. To capture the complexity of social relations, it is beneficial to consider both the structural (e.g., reachability of social network and social integration) and functional (e.g., instrumental and emotional support) aspects of the concept. Both aspects are rarely investigated simultaneously. This study aimed to examine the association between the structural and functional aspects of social relations and poor mental health.

**Methods:**

The study was designed as a cross-sectional register study. We used data on mental health and social relations from 15,839 individuals aged 16–92 years with a mean age of 49.0 years (SD 17.9) who responded to The North Denmark Region Health Survey 2013 among residents in Northern Jutland, Denmark. The 12-Item Short-Form Health Survey measured mental health; a cut-off point of 44.5 was used to dichotomize participants into poor and good mental health. The categorization of social relations was inspired by Berkman et al.’s conceptual model of social relations and health. The analyses were performed with survey logistic regression.

**Results:**

We found that 21.6% (*n* = 3422) of participants reported poor mental health, and 59% (*n* = 2020) of these were women. Being in contact with family and friends less than once a month statistically significantly increased the risk for poor mental health (Family OR = 1.78, 95% CI = 1.51–2.10 and Friends OR = 2.65, 95% CI = 2.30–3.06). The individuals who were not in contact with their network as often as they liked had a significantly higher risk for poor mental health (OR = 2.40, 95% CI = 2.20–2.62). Lack of instrumental support was associated with a higher risk for poor mental health (OR = 2.81, 95% CI = 2.26–3.48). We found an interaction between age and emotional support; the youngest population had the highest risk for poor mental health when they did not have access to emotional support (Young OR = 5.26, 95% CI = 3.91–7.09; Adult OR = 3.69, 95% CI = 3.17–4.30; and Elderly OR = 2.73, 95% CI = 2.23–3.34).

**Conclusions:**

Both structural and functional aspects of social relations were associated with poor mental health in our study. Rarely being in contact with friends and a lack of network reachability were associated with poor mental health. Likewise, low levels of emotional and instrumental support were associated with poor mental health.

**Electronic supplementary material:**

The online version of this article (10.1186/s12889-017-4871-x) contains supplementary material, which is available to authorized users.

## Background

The influence of social relations on mental health has attracted increased interest in recent years [1, 2], as studies have reported an elevated risk of poor mental health among individuals with limited social relations [3, 4]. WHO defines mental health as a state of *“well-being in which every individual realizes his or her own potential, can cope with the normal stresses of life, can work productively and fruitfully, and is able to make a contribution to her or his community.”* [5]. The World Health Organization (WHO) has underlined the need for preventing poor mental health because it is estimated to be one of the major global burdens of disease [6, 7]. Social relations include both structural (e.g., social networks and social integration) and functional aspects (e.g., social support), which influence mental health through different pathways [1, 8, 9]. As the definitions of social relations and methods of measuring relations vary greatly [10], it is difficult to draw firm conclusions about social relations, their effects on mental health, and their universality [10–12]. Berkman et al. considered the impact of social relations on health as a *cascading causal process*. The social context affects the network structures (i.e. network size, range, density, reachability), which influence mental health through different psychosocial mechanisms (i.e. social support, social influence, social engagement). Thus, social support is only one of multiple pathways by which the social networks influence mental health. Social support is divided into subtypes that can include emotional and instrumental support. Emotional support refers to the love and caring provided by significant others, while instrumental support relates to help with practical problems. These psychosocial mechanisms, by which social networks may operate, affect other downstream pathways including health behavioural, psychological and physiological pathways. These three paths, most proximate to the health outcome, may as well be involved simultaneously [1]. This implies that the mental health of individuals depends on how often the individual are in contact with family and friends and the accessibility of social relations, which affects the emotional and instrumental support. This can be understood as a cascade of processes by which social relations influence mental health [9].

The influence of social relations differs by gender [2, 10, 13]. Men and women have different needs for emotional and instrumental support [14]; social support was more beneficial to women than men [14]. A recent systematic review indicated that sources of relations varied over a lifetime and that different age groups had different social relation needs in terms of how much support the individuals needed and from whom the support should be provided [15]. Those with a low socioeconomic position were at a higher risk of poor mental health [16, 17]. The effects on mental health depend on the type of network, such as if the support was provided by family or friends, who can be both a source of support and strain [4, 13].

Prior studies have primarily focused on the influence of either structural or functional aspects of social relations on mental health [9, 11]. Including both structural and functional aspects in studies showed that diverse, quality networks were related to better mental health and may help to protect against poor mental health [4, 8, 10]. The effects on mental health were further related to the type of network; for example, spouses and children had a greater influence on mental health than relatives and friends [8, 10]*.* These findings indicated that both aspects of social relations influenced mental health. As also seen in other studies, the results were limited by small subgroups, such as older adults, decreasing their generalizability [4, 8, 11–14, 17–20].

From a broader population perspective, there is a need to further investigate the association between social relations and poor mental health with consideration for how age, sex and socioeconomic status influence the association between social relations and poor mental health. There seems to be a lack of knowledge of the complexity of social relations and the impact on the individual’s health. To improve understanding of the complexity of social relations and the association between social relations and poor mental health, we examined the association between both structural and functional aspects of social relations and poor mental health.

## Methods

### Study design and setting

The study was designed as a cross-sectional register study and based on data from the North Denmark Region Health Survey 2013 administered and distributed by the North Denmark Region. The survey generated data on health behaviour, self-reported health, social relations, morbidity, etc., and it was used for planning and research in public health [21]. The North Denmark Region is one of five Danish regions; at the time of data collection, the population size was 580,272 citizens [22].

### Participants

The questionnaire was randomly sent to a municipality-stratified sample of 35,700 citizens in Northern Jutland, Denmark, who were aged 16 years and over. Data were collected from the 30th of January 2013 to 1st of May 2013, during which two reminders were sent. The participants could reply by post or online; 20,220 responded to the survey, corresponding to a response rate of 56.6% [21].

**Fig. 1:**
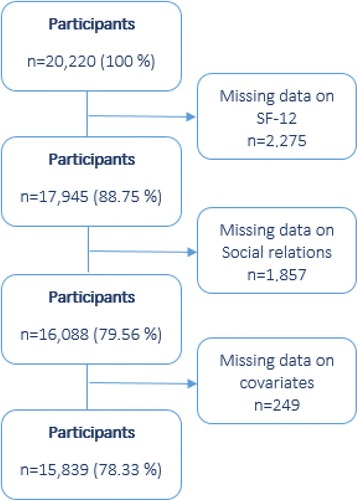
Flowchart. Flowchart of the selection of participants. The right column shows the number (n) of excluded participants

To be eligible for the present study, participants should have answered questions on social relations and mental health. Only participants with no missing answers for all dependent and independent variables were included in the final sample, resulting in 15,839 participants. The study population and exclusion due to missing data are illustrated in a flowchart (Fig. 1).

### Data sources

All people residing in Denmark receive a unique ten-digit Civil Personal Register (CPR) number [23, 24]. Data from the health survey were linked with five administrative registers. The Central Population Register contains information about age, gender, etc. [25]. The Population’s Education register contains information on the individual’s highest completed education [26]. The Danish Register for Evaluation of Marginalization (DREAM), a longitudinal database, includes information on the place of employment and all people who have received government transfer payment [27]. The Income Statistic Register includes all people who are economically active and contains information on the salaries, capital income, private income, etc. [28]. The Danish National Prescription Registry has data on all prescription drugs [29].

### Dependent variable - mental health

Self-rated mental health was measured by the 12-Item Short-Form Health Survey version 2 (SF-12v2), which describes a person’s health condition over a 4-week period [30, 31]. The score from all 12 items of a Mental Component Summary (MCS), ranging from 0 to 100, was calculated [32]. A cut-off point was estimated to differentiate between poor and good mental health, where 100 was the best mental health status [31, 32]. As recommended in the User’s Manual for the Sf-12v2 Health Survey, a dichotomy country-specific, norm-based score was used to establish a cut-off point for ‘poor mental health’ [33]. The cut-off point was set as the mean minus one standard deviation, corresponding to a score of 44.5. Scores above this were considered ‘good mental health’ [34]. Sensitivity analyses were performed with mental health as a continuous measure and further with the United States (US) mean and standard deviation from the SF-12v2 user manual, as suggested by Gandek et al. (cut-off point 40.0) [34].

### Independent variable – Social relations

**Fig. 2:**
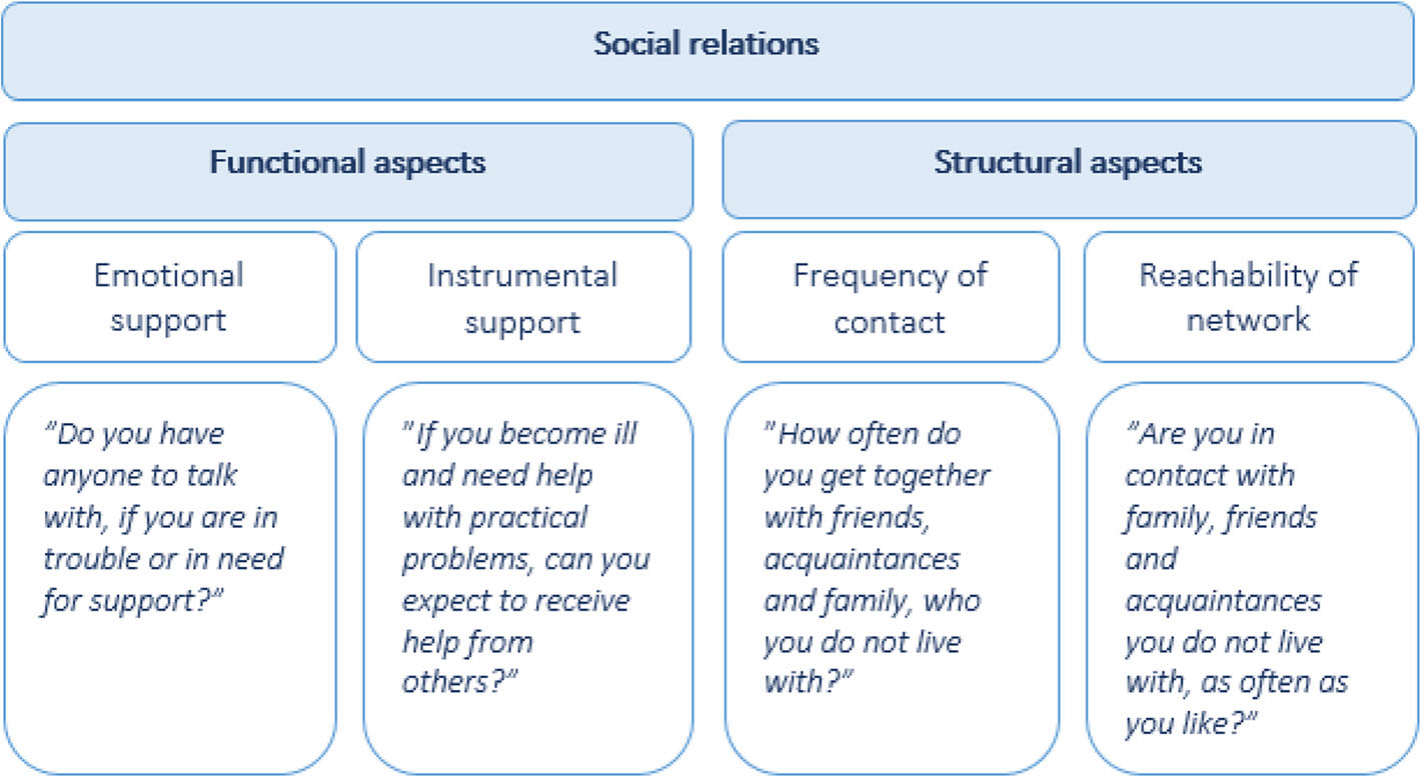
Social relations. Inspired by Berkman et al.’s model [9], the social relations were divided into two groups, functional and structural aspects of social relations. Each question from the health survey was evaluated according to the model. For the question “*Are you in contact with family, friends and acquaintances you do not live with, as often as you like?”* five items were listed, including family, friends, colleagues, neighbours and online friends. The questionnaire was originally in Danish and was translated by the authors of this article

Information on the participants’ social relations was based on self-reported data from the health questionnaire. The questions were classified into functional and structural aspects of social relations inspired by the conceptual model by Berkman et al. [9] (Fig. 2). The frequency of contact was categorized as ‘more than once a week’, ‘more than once a month’ and ‘less than once a month’. Emotional support, instrumental support and reachability of social network (contact with other as often as the individual like) were dichotomized; i.e. yes for emotional support and no for lack of emotional support (Additional file 1).

### Covariates

Age, sex, marital status, ethnicity, socioeconomic status (SES) and depression were, based on the literature, anticipated to influence mental health and were included as covariates [2, 10, 13–17]. Age was classified into three groups, young (16–29 years), adults (30–59 years), and elderly (≤60 years). Age cut-off was selected prior to analysis. The cut-off at 30 years was to ensure that all older had completed education. The cut-off at 60 was when the youngest start to retire. Ethnicity was dichotomized into ‘Danish citizenship’ and ‘other citizenship’; the latter included both western and non-western inhabitants. Information on marital status was obtained from DREAM in 2014 and dichotomized into married or not married and did not include other types of partnership than marriage. SES was measured by education, labour market attachment and income. Educational levels were managed by the United Nations Educational Scientific and Cultural Organization (UNESCO) guidelines for classifying education, the International Standard Classification of Education ISCED (2011) [35]. Participants undergoing education were coded by their highest completed education at the time of response*.* Labour market attachment was based on information at the time the questionnaire was completed and was classified into the following five groups: Employed, Student, Retired (includes retirement, early retirement benefit and voluntary redundancy), Unemployed and Health Related Benefits [36]. Based on an average household income for a 3 year period, four income groups were allocated according to quartiles, 1: 0–35,340 EUR, 2: 35,341–62,596 EUR, 3: 62,597–92,488 EUR, and 4: <92,488 EUR. Participants were classified as depressed if they had redeemed prescriptions for antidepressants (N06A) in the past 5 years [37].

## Statistical methods

Descriptive statistics were performed with the Chi^2^-test to examine the association between covariates and variables for exposure and outcome. *P* < 0.05% was considered statistically significant. Inhabitants living in large municipalities were less likely to be selected for the survey compared with inhabitants living in small municipalities; therefore, data were analysed using the SAS survey logistic regression analysis and presented as the OR and 95% confidence intervals (CI). In the logistic regression analysis age was included as a continuous variable. Prespecified interactions between aspects of social relations and age and gender were conducted and stratified analyses were conducted for significant relations. Sensitivity analyses were conducted to examine the consequences of changing the cut-off points for the MCS score in the survey logistic regression analyses. Sensitivity analysis with mental health as a continues measure were analysed by using analyses of variance.

## Results

### Participants’ characteristics

Table 1 shows the characteristics of the study population by self-reported good or poor mental health. Participants with poor mental health had an average age of 49.0 and were most often women. Compared with individuals with good mental health, individuals with poor mental health were more likely to be depressed, have low income and receive health-related benefits.

**Table 1:** Characteristics of the study population by self-reported good or poor mental health

	Mental Health	
	Good	Poor
N (%)	12,417 (78.4)	3422 (21.6)
Age (mean (SD))	51.4 (17.0)	49.0 (17.8)
Sex, n (%)		
Women	6234 (50.2)	2020 (59.0)
Marital status, n (%)		
Married	7711 (62.1)	1682 (49.2)
Citizenship, n (%)		
Danish	12,147 (97.8)	3276 (95.7)
Education, n (%)		
Basic school	3002 (24.2)	1027 (30.0)
High school	767 (6.2)	268 (7.8)
Vocational education	5164 (41.6)	1286 (37.6)
Short/medium education	2751 (22.2)	651 (19.0)
Higher education	733 (5.9)	190 (5.6)
Labour, n (%)		
Employed	7218 (58.1)	1515 (44.3)
Student	771 (6.2)	298 (8.7)
Retired	3300 (26.6)	722 (21.1)
Unemployed	392 (3.2)	163 (4.8)
Health-Related Benefits	736 (5.9)	724 (21.2)
Income, n (%)		
0–35,340 EUR	5274 (42.5)	1897 (55.4)
35,341–62,596 EUR	5573 (44.9)	1309 (38.3)
62,597–92,488 EUR	1167 (9.4)	165 (4.8)
< 92,489 EUR	403 (3.2)	51 (1.5)
Depression, n (%)		
Depressed	11,608 (93.5)	2615 (76.4)

All *p*-values were below 0.05

**Fig. 3:**
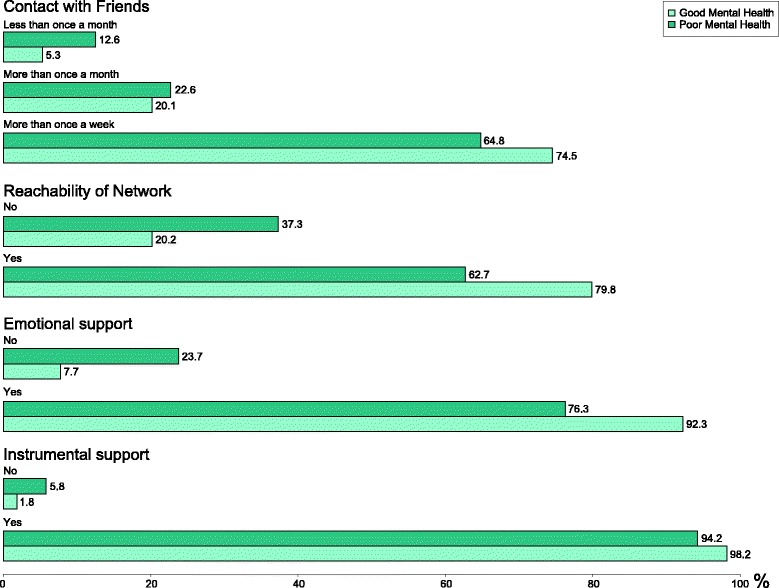
Distribution of selected aspects of social relations

Regarding social relations (Table 2), individuals with poor mental health had less contact with family, friends, colleagues, neighbours and online friends compared to individuals with good mental health, and they were not in contact with people as often as they liked. A higher proportion of those with poor mental health did not have emotional or instrumental support compared to those with good mental health. Based on the level of increased odds for poor mental health, the distribution of selected aspects of social relations is shown in Fig. 3.

**Table 2:** Characteristics of the study populations by self-reported good or poor mental health regarding exposure variables

	Mental Health	
	Good	Poor
n (%)	12,417 (78.4)	3422 (21.6)
Contact with family, n (%)		
more than once a week	10,049 (80.9)	2578 (75.3)
more than once a month	1832 (14.8)	562 (16.4)
less than once a month	536 (4.3)	282 (8.2)
Contact with friends, n (%)		
more than once a week	9252 (74.5)	2217 (64.8)
more than once a month	2502 (20.1)	773 (22.6)
less than once a month	663 (5.3)	432 (12.6)
Contact with colleagues, n (%)		
more than once a week	4068 (32.8)	861 (25.2)
more than once a month	2583 (20.8)	589 (17.2)
less than once a month	5766 (46.4)	1972 (57.6)
Contact with neighbours, n (%)		
more than once a week	6519 (52.5)	1434 (41.9)
more than once a month	2898 (23.3)	694 (20.3)
less than once a month	3000 (24.2)	1294 (37.8)
Contact with online friends, n (%)		
more than once a week	1911 (15.4)	617 (18.0)
more than once a month	1181 (9.5)	300 (8.8)
less than once a month	9325 (75.1)	2505 (73.2)
Reachability, n (%)		
Yes	9914 (79.8)	2144 (62.7)
No	2503 (20.2)	1278 (37.3)
Emotional support, n (%)		
Yes	11,457 (92.3)	2611 (76.3)
No	960 (7.7)	811 (23.7)
Instrumental support, n (%)		
Yes	12,191 (98.2)	3225 (94.2)
No	226 (1.8)	197 (5.8)

All *p*-values were below 0.05

### Social relations and mental health

**Fig. 4:**
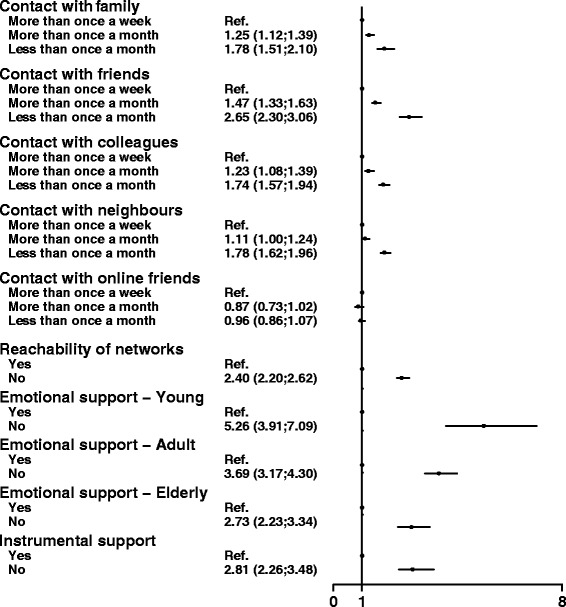
Survey logistic regression model showing the adjusted estimate between social relations and poor mental health. Model 4. Adjusted for age, sex, marital status, ethnicity, SES (education, occupation and income) and depression

Associations between aspects of social relations and risk of poor mental health adjusted for relevant confounders are shown in Fig. 4. Univariate analyses were nearly identical to the multivariable analyses; therefore, only the fully adjusted results are shown. The importance of emotional support depended on the age and is therefore presented in three age groups (*p*-value for interaction <0.01).

#### Structural aspects of social relations

As shown in Fig. 4, a low frequency of contact with family, friends and acquaintances increased the risk for poor mental health. Spending time with family and friends more than once a month statistically significantly increased the risk for poor mental health compared to being together more than once a week. There was no statistically significant difference between spending with colleagues or neighbours more than once a month and more than once a week.

Spending time with family, friends, colleagues and neighbours less than once a month statistical significantly increased the odds for poor mental health compared to spending time together more than once a week. Lack of reachability of networks statistical significantly increased the odds for poor mental health. There were no statistical significant associations between contact with online friends and poor mental health.

#### Functional aspects of social relations

Absence of instrumental support statistically significantly increased the odds for poor mental health. Higher odds for poor mental health remained throughout covariate adjustment.

Emotional support was associated with age. The stratified analysis (included in Fig. 4) showed a statistically significant increase in the odds for poor mental health among all age groups, and the youngest participants had the highest risk for poor mental health.

#### Sensitivity analyses

The sensitivity analyses with mental health as a continuous measure produced similar results as the main analysis in model 4. Being together with friends less than once a month statistically significant decreased the score of mental health (−4.79, 95% CI = −5.44;-4.43). Lack of reachability decreased the score of mental health (−4.09, 95% CI = −4.45;-3.74). Lack of instrumental support and emotional support likewise decreased the score of mental health (−5.23, 95% CI = −6.32;-4.14) and (Young: -8.80, 95% CI = −10.38;-7.22, Adult: -6.58, 95% CI = −7.27;-5.90, Elderly: -4.14 95% CI = −6.32;-4.14), respectively.

Changing the cut-off point for the US mean and standard deviation (cut-off point 40.0) on all models produced similar results as those in the main analysis.

## Discussion

Overall, low levels of social relations were associated with poor mental health in our study. Our findings suggested that the frequency of contact with friends and the reachability of network had a significant influence on poor mental health. Likewise, all functional aspects of social relations, such as emotional and instrumental support, significantly influenced poor mental health.

### Interpretation

Our findings indicated that the odds for poor mental health increased as the individual’s frequency of contact with family, friends, colleagues and neighbours decreased. This corresponds with previous studies that found that mental health was influenced by the frequency of contact with different kinds of networks [8, 38]. Those with self-reported good mental health had more frequent contact than those with poor mental health. Based on our results, poor mental health may depend more on the frequency of contact with friends than with family. This can be a consequence of different networks providing different types of support. Furthermore, friendship is often characterized by interchangeability, where friends, who can contribute to strains, can be excluded from the individuals’ network [10]. The findings in this study indicated that those who were not in contact with their family, friends and acquaintances as often as they liked had twice the risk of poor mental health compared to those who were in contact as often as they liked. Therefore, it is possible that the association between low levels of contact and mental health depend on whether the frequency of contact corresponds to the individual’s need for contact.

In agreement with other studies, the present study found a higher prevalence of women with self-reported poor mental health than men [17], but our findings did not support the influence of gender. A possible explanation is that while social relations are essential for both genders, different aspects of social relations can have different meanings across genders, which was also found by Almquist et al. and Fiori et al. [13, 14]. One fourth of participants with poor mental health in our study lacked emotional support. In particular, young individuals had a higher risk for poor mental health if they did not receive emotional support. From a life course perspective, it has been suggested that the individual is more dependent on social relations in some stages of life than in others [2, 9]. We did not find interactions between age and other measures for social relations, which suggest that, regardless of age, social relations have a significant influence on poor mental health.

A lack of instrumental and emotional support resulted in an approximately threefold increased risk of poor mental health. This finding of a strong association between instrumental and emotional support and poor mental health is consistent with other studies [4, 9, 10, 39, 40]. This may be because the support enables the individual to overcome challenges and instrumental and emotional support strengthens coping strategies [9]. These findings underline the need for a reachable social network where individuals have the opportunity for contact others as often as they need. Furthermore, the relations should provide both instrumental and emotional support. The findings underline the importance of both structural and functional aspects of social relations on mental health, as emphasized by Berkman et al. [9].

Individuals with poor mental health have an increased risk for mental disorders, such as depression [5, 41]. Therefore, there is a close relation between poor mental health and depression [42]. We had a low tolerance for classifying individuals as depressed, and while depression is the major reason for using antidepressant these drugs are also used for anxiety. Consequently, 10% of the study population was classified as depressed, while the prevalence in Europe was 6,9% [43] and it was 4,1% in the Danish population [44]. Therefore, the estimates for poor mental health where adjusted for depression to ensure that estimates correspond to poor mental health, although adjustment may have caused an underestimation of the association between social relations and poor mental health.

### Limitations and strengths

A strength of this study was the large sample size and the use of nationwide registers, providing high quality data and reliable information on a wide range of covariates, which enabled reliable, comprehensive confounder adjustment [45]. In addition, we could use self-reported data on social relations and mental health. The individual’s perceptions of social relations and mental health are essential to understanding the association because both aspects are characterized by the individual’s experience. This is underlined by the WHO definition of mental health as *“a state of well-being in which every individual realize his or her own potential, can cope with the normal stresses of life, can work productively and fruitfully, and is able to make a contribution to her or his community”* [5].

The cross-sectional design of the study excluded conclusions about causality, i.e. poor mental health causing less contact with family, friends and acquaintances. The use of the survey logistic regression reduced potential selection bias from unequal selection in each municipality [46–48]. Non-response can contribute to the risk of selection bias, where people with poor mental health are assumed to participate less in a survey, which can cause an underestimation of the association [17]. Respondents were more likely women, middle-aged, native, and married and they had a higher education and good mental health [21, 49–51].

We were inspired by Berkman et al.’s conceptual model that incorporates both structural and functional aspects of social relations. Our use of a narrow selection of measures for social relations may be a limitation because the use of a few measures is less likely to capture the full complexity of an individual’s social relations. The specific social relations-related questions asked in the health study have been used for several years in major Danish health surveys [21, 52] and to answer similar questions in international studies [10]. However, there is a need for further development and validation of a scale to measure structural and functional aspects of social relations.

The SF-12 is a reliable, validated measurement based on the SF-36 Health Survey [30, 33]. Other studies have validated the SF-12 in both general and specific populations. In a Danish setting, the SF-12 was validated in 2012, and it was documented as a reliable, valid instrument for measuring health-related quality of life [30]. The dichotomization of mental health followed the recommendations from the SF 12 user’s manual, which strengthened the generalizability of our results. The sensitivity analysis based on the US standard cut-off (40.0) did not alter the results; therefore, it is likely that our results can be compared and interpreted across populations [34]. However, the dichotomization of the mental health score reduced the statistical power [53] and the nuances of mental health.

### Implications for research and practise

This study adds to the existing knowledge about the association between social relations and mental health. Mental health depends on the frequency and type of interactions with others and to what degree a person’s network of friends and family is reachable; a lack of all aspects of social relations is a risk factor for poor mental health. In the prevention of poor mental health, it is important to consider a broader perspective on social relations to capture their complexity. There is a need for further research on how both aspects can be included in interventions that target mental health.

## Conclusion

The present study demonstrated that both structural and functional aspects of social relations were associated with poor mental health among citizens in Northern Jutland, Denmark. Poor mental health was more prevalent among the young and adults. Our study indicated that the odds for poor mental health increased as the individual’s frequency of contact with family, friends, colleagues and neighbours diminished. The young individuals who lacked emotional support were at the highest risk for poor mental health. The study contributes to the understanding of the complexity of social relations and their effect on the mental health of individuals and claim attention to avoiding simplifying the mechanisms behind social relations. It underlines the importance of an understanding of the complexity of social relations and the need for interventions aimed at preventing poor mental health as well as further research to consider both structural and functional aspects of social relations.

## Additional file

Additional file 1: Questionnaire regarding social relations; translated by the authors of the present study. (PDF 758 kb)

## Abbreviations

CI: Confidence intervals
CPR: Civil Personal Register
DREAM: The Danish Register for Evaluation of Marginalization
ISCED: International Standard Classification of Education
MCS: Mental Component Summary
SD: Standard Deviation
SES: Socioeconomic status
SF-12v2: Short-Form Health Survey version 2
UNESCO: United Nations Educational Scientific and Cultural Organization
US: United States
WHO: World Health Organization
