# Supplementary material for: Associations between the structural and functional aspects of social relations and poor mental health: a cross-sectional register study
Source: BMC Public Health. 2017 Nov 3;17:860. doi: 10.1186/s12889-017-4871-x (PMC5670730; doi:10.1186/s12889-017-4871-x)
Supplement: Additional file 1: — Questionnaire regarding social relations; translated by the authors of the present study. (PDF 758 kb) [file 12889_2017_4871_MOESM1_ESM.pdf]

Additional file 1. These questions were translated by the authors of the present study. The original file in Danish can be found at http://www.rn.dk/Sundhed/Til-sundhedsfaglige-og-samarbejdspartnere/Folkesundhed/Publikationer

How often do you get together with friends, acquaintances and family, who you do not live with?
(Contact meaning that you are together, talking on the phone, writing to each other, etc.)

(set one X in each line)	**Daily or almost daily**	**1 or 2 times a week**	**1 or 2 times a month**	**Less than 1 time a month**	**Never**
Family you do not live with	❑	❑	❑	❑	❑
Friends	❑	❑	❑	❑	❑
Colleagues or fellow students	❑	❑	❑	❑	❑
Neighbours or residents in your local community	❑	❑	❑	❑	❑
Persons mostly known from internet (mail, chat etc.)	❑	❑	❑	❑	❑

Are you in contact with family, friends and acquaintances you do not live with, as often as you like?
(Contact meaning that that you are together, talking on the phone, writing to each other, etc.)

	(Only one X)
Yes	❑
No	❑

Do you have anyone to talk to, if you are in trouble or in need of support?

	(Only one X)
Yes, often	❑
Yes, mostly	❑
Yes, sometimes	❑
No, never or almost never	❑

If you become ill and need help with practical problems (e.g. cooking, shopping, cleaning, dressing), can you expect to receive help from others?

	(Only one X)
Yes, definitely	❑
Yes, maybe	❑
No	❑
Do not know	❑
